# Mr. Whiter's Dissertation on the Disorder of Death, or That State of the Frame Termed—"Suspended Animation"

**Published:** 1821-03-01

**Authors:** 


*A Dissertation on the Disorder of Death; or that state of the Frame under the Signs of Death, called "Suspended Animation," to which Remedies have been sometimes successfully applied, as in other Disorders; in which it is recommended, that the same Remedies of the resuscitative Process should be applied to Cases of Natural Death, as they are to Cases of Violent Death, Drowning, &c.* By the Reverend WALTER WHITER, Rector of Hardingham, Norfolk, and late Fellow of Clare Hall, Cambridge. One vol. 8vo, pp. 480. London, 1819.

> "Death may usurp on Nature many hours,
> "And yet the fire of life kindle again
> "The o'erpressed spirits." *Pericles.*

WHATEVER difference of opinion may exist among philosophers, metaphysicians, and divines, respecting man's probable destiny hereafter, all seem agreed that he is placed here for some wise purpose, whether finite or probationary; and that whatever suddenly arrests or slowly curtails the natural range of his existence, is an evil of the greatest magnitude, and one that is to be obviated by all possible means. Sudden death is not only so revolting to human nature, but so shocking and distressing to surrounding relatives, that Heaven has kindly ordained the remembrance of it to be transitory—unless where its consequences entail lasting misery on the survivors, and too indelibly engrave it on the tablets of their memory. It must be confessed, that all our investigations into the nature of life—that Being which we are so anxious to preserve, and so unwilling to resign, have ended in disappointment, or only portrayed to us the extent of our ignorance, and the futility of the pursuit. That great secret of our Creator yet mocks our speculations—eludes our search—and, like the rainbow or horizon, "still as we follow, flies."

"We are amazed and confounded" says the learned and zealous writer of the work before us, "when we contemplate the union of thought, or even of motion and sensation, with organized matter in the functions of animal life; and our amazement ought still perhaps to be increased, when we meditate on that condition of the frame, in which this union appears at once to be dissolved, and the action of the vital principle is visible no more." P. 2.

It is but too well known, that we have no definite criterion (anterior to putrefaction) between animation *suspended*, (consequently within the possible range of revival) and animation *extinct*. Modern experience has exhibited extraordinary facts which have overturned, in many cases, the conceptions of all preceding times, on the subject of life and death;—and they may suggest to us, that other cases of erroneous, or perhaps pernicious opinions, are still in too general operation. We live in an æra when, without a miracle, the *dead* have been raised to *life* and all the blessings of existence. We have ascertained that motion and sensation are *not* necessary indications of the presence of vitality—and, finally, that "the powers of *life* may remain, though the signs of *death*, according to all former notions on the subject, may be unequivocally apparent." The most successful and brilliant proofs of this fact have been exhibited by the Humane Societies—proofs which have overturned the *experience*, as it has been called, of all former ages, in every region of the globe.

"It is now acknowledged, that the most illustrious sages in the art of medicine and the grossest of the people, in all former times, have gazed with the same eye of fatal and unsuspecting ignorance on various appearances of *Death*; and that they have committed their fellow creatures to the grave, who were still instinct with the principles of *Life*, and possessed even with the powers of a sound and healthy frame, ready at once to resume in all its former vigour, the various offices of motion—sensation—and reflection." 9.

This brings us to make a few observations on the work before us and its author. We think we may safely conclude, that the great body of the profession, like ourselves, have hitherto been prepossessed against, or rather careless about, the work in question; partly from the nature of the title page, and partly from the extra-professional character of its author. We were lately induced to peruse the publication, by the recommendation of some friends, and in it we found much to interest our feelings—much to amuse our minds—and much to improve our understanding, even in a professional point of view. Mr. Whiter has been unfortunate—we had almost said, injudicious, in two things—first, in refining, we humbly conceive unnecessarily, on what is commonly understood, in the profession, by the term "suspended animation," and giving it the uncouth appellation—"disorder of death," which, after all, means neither more nor less than that state of apparent death within the possible range of reanimation—in plain terms—"suspended animation." It is true that our author has clearly and unequivocally explained his meaning; but then it is at the expense of reading several pages—an expense that can be worse spared than money in this biblical age, when nearly as many books issue from the press, as human beings from the wombs of their mothers. The greater misfortune is, the general prolixity in which Mr. Whiter has indulged—evidently with the laudable *motive* of impressing more strongly his reader's mind with the importance of the subject, and the justice of his cause; but, we fear, too often with the *effect* of weakening the one, and rendering suspected the other. There is, moreover, no doubt in our minds, that our author has, unintentionally, exaggerated the extent of the evil which he laments, and formed too sanguine expectations of the remedy which he would apply. Still there is a vast deal of good sense, humane feeling, and sound reasoning, throughout the work —and quite enough to repay the labour of perusal.

We cannot follow Mr. Whiter through his long chain of argumentative reasoning; but rather endeavour to show its tendency or purport. Our ingenious author thinks that the experience of the Royal Humane Societies has not supplied us with any new principles by which we can infallibly decide on the irrecoverable annihilation of the vital powers. On the contrary, it appears "that the only evidence respecting the total extinction of life, is to be found in that mode of decision to which they have themselves resorted in those peculiar instances which they have undertaken to examine." As this evidence consists in actual experiment, nothing, he thinks, but the ineffectual trial of the resuscitative process can authorize us to pronounce the sentence of *absolute and irremediable* death.

The "disorder of death," or suspension of the vital powers and processes, is the most terrific of all disorders, "as it almost invariably terminates in final and putrefactive dissolution," *where no remedies are applied to its relief.* And here our author very forcibly draws our attention to the fact that, although the transactions of Humane Societies directly conduct the understanding to the above melancholy inference, yet a conclusion so obvious has had no operative effect either on the minds of these societies, or on the opinions of the public to whom they have appealed.

"The truth which they have established, remains an insulated fact, and confined within the narrow limits of the particular cases, by which it has been proved. It has not been adopted as the basis of a general conclusion, nor has it operated on the customs and institutions of mankind. In vain have these Societies demonstrated to the most perfect satisfaction of themselves and the public, that the absence of apparent motion and sensation affords *no* criterion whatever of the annihilation of Life. The artists, who have exhibited the most numerous and brilliant proofs of this truth, and the people, who have been convinced by the demonstration, still continue to regulate their ordinary practice by a maxim directly opposite, just as if no such Societies had existed, and no such truth had been established. The absence *of apparent motion and sensation* still continues in these times as in all the past, to be regarded in our ordinary practice as an *infallible criterion of the annihilation of Life;* and on this conclusion at this moment, through the whole extent of the globe, we consign our fellow creatures to their graves, without reflection or remorse." 13,

If this suspension of vitality, or "disorder of death," then, has been sometimes cured by the resources of art, the question is, whether we should not, *in all other cases*, (as well as in those of drowning or sudden death) employ the resuscitative process? Our author answers decidedly in the affirmative.

"It is the purpose of this Treatise to urge in the most strong and unqualified terms, that the process of attempting to revive suspended animation, which has been adopted only under particular accidents, should be applied to *all cases of death, under all circumstances, and upon all occasions.*" 34.

In the next page our author qualifies, in some degree, the extent of this principle, by stating that he means by "*all* circumstances and *all* occasions," only to propose "that the remedies should be as *universally* applied in this *disorder*, as they are in all other diseases, by which the frame may be attacked."

The professional reader, especially the practical physician, acquainted with the discoveries of modern pathology, will strongly object even to this limited application of the principle in question. A man dies, worn to a skeleton, with hectic fever and purulent expectoration. The attendant medical practitioner thinks it his duty, no doubt, to administer remedies till the last—not with the hope of cure, but as palliative of distressing symptoms. Or a man dies with all the characters of some great internal aneurism. Who would think of attempting the resuscitative process in these and hundreds of other cases, where vitality ceases in consequence of organic changes in important viscera. Indeed, the author himself, a little farther on, narrows his principle still farther, and obviates substantially the objection which lies against his first broad and sweeping proposition.

> "His projects are not designed to recal the principle of vitality, when it is *altogether* decayed or exhausted, but to *revive and retain its power*, while it remains surrounded by the materials, either sound or not violently impaired, which are the instruments of its action." 36.

While it unfortunately happens, in by far the greater number of fatal cases, that some part of the material fabric is seriously impaired; still we must bear in mind how frequently we see the thread of life, suddenly and unexpectedly, snapped, when no warning symptom appeared *before*, nor necessarily fatal disorganization *after*, death. How often, for instance, during convalescence from grave, acute diseases, do we, with surprize, learn the sudden death of our patient; and, after a strict *post mortem* examination discover not a trace of organic lesion, beyond perhaps some turgidity of vessels that might or might not have any thing to do with the unexpected arrest of vitality. How are we certain that, in such circumstances, the resuscitative process promptly applied, might not have re-kindled the vital spark?—It is to this class of sudden and *unexpected* deaths, during and subsequent to other diseases, as well as to accidents in health, that we think the force of Mr. Whiter's arguments is mainly applicable; and he will, in future ages, be deemed no mean benefactor to the human race, if his work shall succeed in rousing the attention of the medical community to this interesting question, and their efforts shall be afterwards crowned with even a very small portion of that success which his wishes may anticipate.

There is much to be said in favour of the attempt, and little or nothing against it.

"In this project, the activity of our zeal does not commence, till the extremity of the evil belonging to the case has been incurred, and when that period is arrived at which no danger is to be feared from the theories of the enthusiast, or the practices of the credulous." 45.

The fear of premature interment has often prevailed, almost epidemically, in this and other countries, founded on the histories, some true, but most of them false, of spontaneous re-animation. It is strange, however, as our author has hinted in various places, that this dread of revival in the *grave*, or hope of such an event before interment, should not have led to the employment of some means to effect resuscitation, si lateat scintillula forsan, and thus obviate the chance of horribly awaking in the grave! The fact is, that the survivors leave the apparent or real corpse to slowly change into putrefaction, without giving Nature the slightest assistance in resisting this process, or reviving the smothered spark of vitality—if a spark should remain.

"The possibility of latent life, which they acknowledge, does not fill their minds, as I observed, with hope, but with horror:—It does not suggest conceptions or devices for the recovery of that life, but it inspires them with the desperate project of preventing such a recovery by its absolute annihilation. Their terrors are not appeased, till they have *killed* the object, in whom they suspect this fearful property of life to be still lurking; nor are their minds composed, till they have made assurance double sure, and converted doubtful apparent death into certain—unequivocal putrefactive death, in which every spark of latent life is for ever extinguished without the possibility of recovery or recal." 65.

Such is the picture which our author has drawn of *protracted* interment; but the hurried inhumations practised by some nations, and particularly the Jews, who we believe bury their dead on the same day they die, has excited Mr. Whiter's imagination to a degree that has, we fear, somewhat obscured his judgment. Instead of considering the grave as the cold receptacle of our mortal remains, that quickly must extinguish any lingering ember of vitality, Mr. Whiter looks upon it in quite another light—"as *the warm genial spot of earth* endowed with every property most propitious to the process of resuscitation"—and, that upon no less authority than one of our "PROFESSIONAL SAGES," the M. D. *i. e.* Dealer in Medicine Chests, of SOUTHCOAT memory, who "has conceived that *precious property* of the ground under a point of view, which induces him to communicate this truth to the public, as an *article* of the most alarming intelligence. He warns the incautious world not to trust the corpse too soon within the *restorative and balmy*

*precincts of the grave*, lest perchance it should recover in a spot which, under his mode of conception, becomes a portentous scene of horrible efficacy in the process of re-animating the dead." 67. Mr. Whiter is not perhaps aware that, in the multifarious writings of the Southcoat "sage," nominal, anonymous, and pseudonymous, there are more absurdities than sentences—more falsehoods in every page of his spurious productions, than hieroglyfics round the walls of this vender of rhatany root, medicine chests, and calumnies. Mr. Whiter is therefore to be pitied and excused for this unfortunate citation, which, without many redeeming qualities, would ruin his work.* We do not consider it necessary to make any comments on this part of our author's work, as the subject is not likely to be productive of any advantage. That the inhumation of our fellow creatures, before the vital heat has had time to leave the body, is to be censured and condemned, no one will deny; but that the earth, thrown up out of the grave, and lowered to the temperature of all surrounding bodies, should, when thrown in on a corpse, defended by shroud, coffin, and cover, have any *balmy* or *restorative* effect, can be entertained only by a man who believed in the advent of a young Shiloah, from the dropsical ovaries of Johanna Southcoat, as announced by the ventral rumblings of that flatulent old woman.

We must pass over that division of Mr. Whiter's work which treats on the "sleep of death, and the death of sleep," though it contains a vast store of curious matter, ingenious reflections, and learned researches. This section, indeed, will be found extremely amusing to all classes of society; but it is quite incapable of analysis.

A considerable portion of the publication under review, is dedicated to "remarks on the treatment of the dying and the dead;" and from this portion we shall extract some matter that may not prove uninteresting to our readers, especially as it proposes—" a new source of comfort for the attainment of a blessing, which is considered as the last good of our earthly condition—the blessing of *Euthanasia*."

---

* For the honour of the Profession we do not know a single medical writer who has condescended to mention this vile excrescence on the noble tree of science, excepting with horror and detestation. The whole course of our reading, and the whole circle of our acquaintance round this terraqueous globe, have not furnished us with a character so truly despicable, as the abovementioned disgrace to the very name of science or literature. Fortunately, as an *author*, he is out of the pale of even the lowest circle of the Profession. As a *man* we know him not.

With Dr. Ferriar, our author reprobates some of the monstrous customs of nurses and undertakers—for instance, the dragging of the bed from under dying persons, and the placing them on a mattress—pulling pillows and bolsters from below their heads—stopping up the inlets of breath, and the outlets of excretions—binding the breast and arms round with a bandage—stripping the body in cold weather, and laying it out in little more than half an hour after sudden death—all of which Dr. Ferriar, and of course, our author, think the helpless patient may be sensible of, though incapable of expressing his sensations distinctly. Our author finds fault with Dr. Ferriar for supposing that after twelve hours of *apparent* death there is little chance of re-animation, lest perchance "the consciousness should be extended beyond this period." We really are completely sceptical as to this *consciousness* during the absence of every external phenomenon of vitality. Can there possibly be consciousness after the heart and lungs have ceased to perform their office, even for a few minutes? Mr. Whiter censures with more justice the direction of Dr. Ferriar to draw the curtains nearly close round the bed of the dying man when the rattling noise of respiration and difficulty of swallowing have come on.

Here, as in many other places, Mr. Whiter reflects on the conduct of the medical profession in abandoning the patient as soon as he is apparently dead, instead of trying resuscitative means; but these reflections grow out of the erroneous or extravagant extent to which our author appears inclined to carry his theory—identifying cases of natural death with those from sudden arrests of life in the midst of health. But here lies the whole difference. In the *latter* cases, means of resuscitation ought undoubtedly to be used; but to extend these means to the *former* class would not indeed prove a cruelty to the dead—but a source of torturing suspense to the feelings of the relatives, without, we fear, being, in any instance, productive of ultimate success. Our author, however, thinks very differently, and counsels thus:

"The signs marked on the *dying* and the *dead* are fallacious. Trust not, I cry, to powers of prediction, which you do not possess: *doubt* and *decide not:* The dying man may be the sinking man, exhausted by his malady, or perhaps exhausting his malady, and fainting under the conflict.—Exert all the arts, which you possess, and which have been found not only able to resuscitate and restore the dying but even the *dead:* Rouse him from this perilous condition, and suffer him not, by your supineness and neglect, to pass into the state of final and putrefactive death." 328.

Under the impression that consciousness may remain for some time after the human frame has ceased to exhibit any of the phenomena of vitality, our author suggests, as an important point of euthanasia, that the expression of those agonizing sorrows attendant on the last scene of life—the lamentations of father, mother, husband, wife, and other relatives, should be banished from the bed of death. "The violence of grief will be then suspended or alleviated, till all the resuscitative arts have been adopted and exhausted in vain. *The dying man departs with the hope of life, and the howl of despair is not raised, till he can hear it no more.*" 333.

It was our intention to have gone much further into the analysis of the work before us than we have done; but the style of the author and his arrangement, or rather want of arrangement of the matter, render it impossible for us to do justice to the work, without expending time, space, and labour, which our other duties will not permit. We trust that we have done enough to excite to the publication the notice of the Profession—a notice which has hitherto been confined to a very limited circle. The humane and philanthropic feelings of the author have raised him very high in our esteem, while the ingenuity of his reasonings, and the extent of his knowledge, command our most unlimited respect.

